# Weight Estimation for Drug Dose Calculations in the Prehospital Setting – A Systematic Review

**DOI:** 10.1017/S1049023X23006027

**Published:** 2023-08

**Authors:** Mike Wells, Brendon Henry, Lara Goldstein

**Affiliations:** 1. Department of Emergency Medicine and Critical Care, Herbert Wertheim College of Medicine, Florida International University, Miami, Florida USA; 2. HCA Florida Aventura Hospital, Aventura, Florida USA

**Keywords:** Broselow tape, drug dosing, PAWPER XL tape, weight estimation

## Abstract

**Background:**

Weight estimation is required to enable dose calculations for weight-based drugs administered during emergency care. The accuracy of the estimation will determine the accuracy of the administered dose. This is an important matter of patient safety. The objective of this systematic review was to collect, review, evaluate, and create a synthesis of the current literature focusing on the accuracy of weight estimation in the prehospital environment.

**Methods:**

This systematic review followed the PRISMA guidelines. Studies were identified and included if they were peer reviewed, full length, published in English, and contained original data. Studies utilizing any form of weight estimation methodology in the prehospital setting (in children or adults) were included. Data on the quality of the studies and accuracy of the weight estimation systems were extracted. Common themes were also identified.

**Results:**

Twenty-five studies met the inclusion criteria, with only nine studies (36.0%) containing useful weight estimation accuracy data. The overall quality of the studies was poor. The Broselow tape and paramedic estimates were the most studied methods of weight estimation, but there was insufficient evidence to support conclusions about accuracy. The major themes identified included the importance of accurate weight estimation and drug dosing as critical matters of patient safety, and the need for training to ensure these processes are performed accurately.

**Conclusions:**

There were limited robust data identified on the accuracy of different weight estimation methods used in the prehospital setting. Future high-quality clinical research in this area is of critical importance to ensure patient safety in the prehospital environment.

## Introduction

The dosing of medications by Emergency Medical Services (EMS) paramedics is often based on weight, especially in children.^1^ There is insufficient time and resources to weigh critically ill or injured patients in the prehospital setting, and having a safe, accurate, and reliable way to estimate weight is an integral step to ensuring positive outcomes.^2^ With medication errors occurring in more than 28% of EMS encounters (when drugs are administered), and with weight estimation errors a significant contributor to these errors, a weight estimation technique that is rapid, accurate, reliable, and easy to use in the prehospital environment is essential.^3^ This would facilitate dose calculations by EMS practitioners and would also allow for the receiving facility to prepare for the patient’s arrival.

Weight estimation techniques in children have generally been well-described in the Emergency Medicine literature. They include parental estimations, visual estimations by health care providers, age-based formulas, length-based methods (such as the Broselow tape), and the newer – and most accurate – length- and habitus-based methods (such as the Mercy method and the PAWPER XL tape).^4^ In adults, the need for weight-based dosing (and therefore, weight estimation) is less common than in children, but it is still important.^5^ Weight estimation methods in adults include self-estimations, estimations by family members, visual estimations by health care providers, anthropometric formulas, automated computerized methods, and the use of pediatric methods in adults.^6^ Patient self-estimations have been shown to be the most accurate, but this might not be possible in an incapacitated patient, and a reliable method of estimation needs to be available.

There is very little known about what weight estimation methods are used in prehospital settings anywhere in the world.^7^ There is less known about how accurate these methods are in the EMS environment. To the authors’ knowledge, there has not been a systematic review on prehospital weight estimation systems and how accurate and reliable the various methods are when used by EMS practitioners. This could be an important source of information to determine the best strategies for estimating weight by EMS practitioners. This information would also be useful for policymakers and protocol boards.

The purpose of this study was to conduct a comprehensive review, analysis, and synthesis of the existing literature on weight estimation practices in the prehospital emergency medical care setting. The specific aims were to evaluate the quality of relevant published research, to identify the weight estimation methods used in prehospital medicine, to assess the evidence supporting the effectiveness and accuracy of the described methods, and to identify important themes arising from the studies.

## Methods

### Identifying Relevant Studies

This systematic review was based on the Preferred Reporting Items for Systematic Reviews and Meta-Analyses (PRISMA) methodology, with a protocol registered with PROSPERO (registration number CRD42021253761). No significant amendments of, or deviations from, the registered protocol were noted. A literature search was conducted for publications from January 1988 through September 2022 using PubMed (National Center for Biotechnology Information, National Institutes of Health; Bethesda, Maryland USA), Embase (Elsevier; Amsterdam, Netherlands), Web of Science (Clarivate Analytics; London, United Kingdom), and Google Scholar (Google Inc.; Mountain View, California USA) databases. The Boolean search terms “weight estimation” OR “weight prediction” AND “prehospital” OR “EMS” OR “Emergency Medical Services” OR “paramedic” OR “out of hospital” were used. Additional searches were done based on the “similar articles” section of PubMed and by reviewing the bibliographies (and the Medical Subject Headings [MeSH] terms) of the papers identified in the searches. To minimize reporting biases, broad inclusion criteria were used, and multiple databases were used for the search. This included searching for studies in the “grey literature.”

### Study Selection

**Figure 1.**
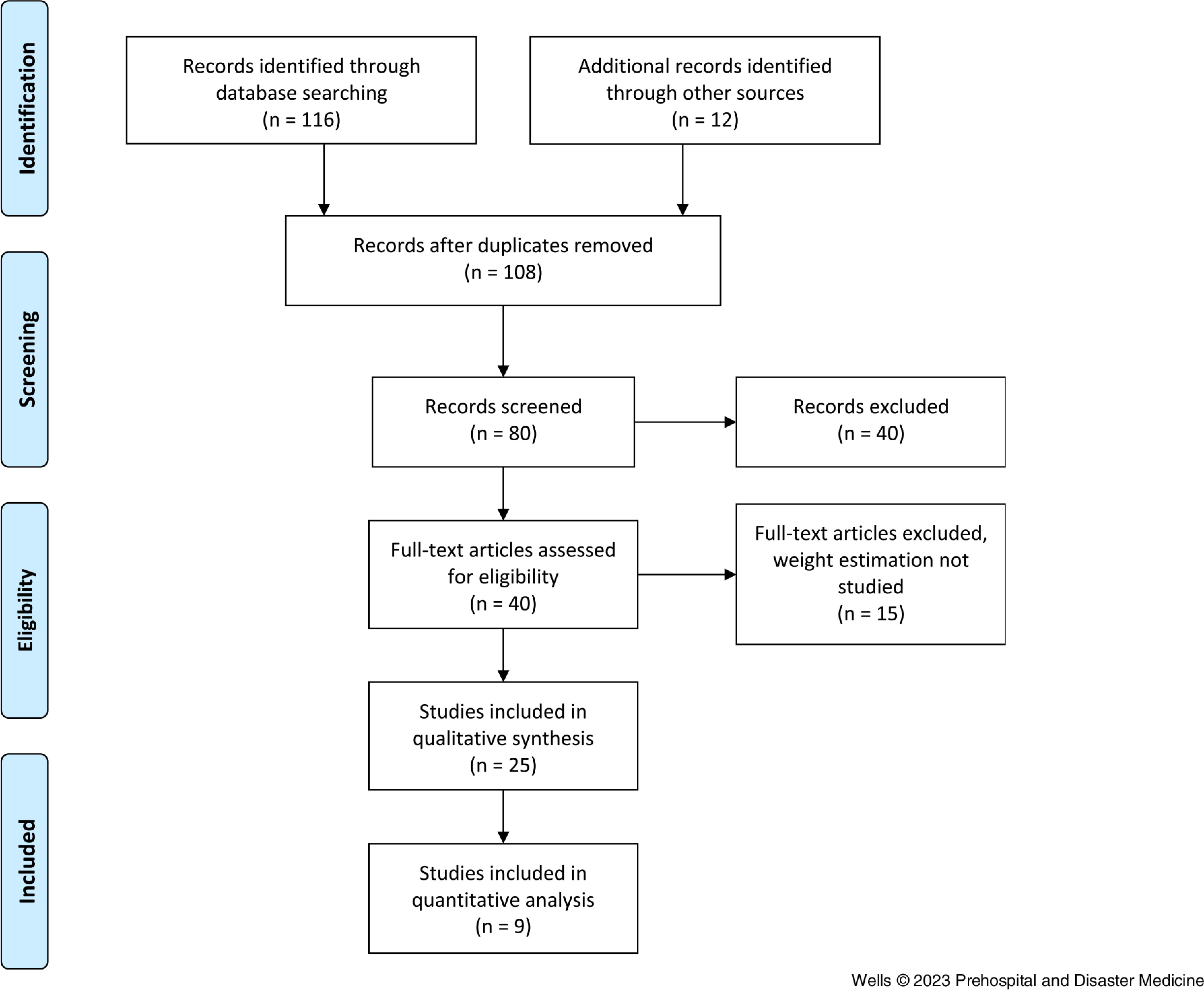
PRISMA Flow Chart for the Identification and Selection of Studies.

Studies were screened if they were peer reviewed, full length, published in English, and contained original data (Figure 1). Studies were considered for inclusion if they reported on weight estimation by any method, in any age group, and were related to EMS in any way. Studies on drug dosing accuracy were included if data pertaining to weight estimation were included. Studies were excluded if there was no reference to weight estimation. Screening was conducted by two researchers independently (BH and MW), and the identified articles were reviewed and screened by the other researcher.

### Data Extraction

The following data were extracted from each included study: study information (publication date, number of patients, origin of study), study design, the methods of weight estimation used, the environment the study was performed in, the age range, the location, and the major findings. The data were extracted by one author (BH) and the accuracy independently confirmed by the other authors (MW, LG).

### Data Analysis and Grading of Evidence

The approach used to assess the certainty of the evidence from the included studies included as assessment of the risk of bias (and study limitations), inconsistency in results (heterogeneity), indirectness of evidence, imprecision and statistical or methodological flaws, and publication bias. Each included study was graded for quality of evidence using a modified Newcastle-Ottawa scale, as has been described previously (Supplementary Table 1; available online only).^4^ Each study could score a minimum of zero stars and a maximum of ten stars on the modified Newcastle-Ottawa scale. An assessment of selective non-reporting or under-reporting of results in the studies was included in the Newcastle-Ottawa scale. On this scale, a study with score from six to ten has high quality, four to five has a moderate risk of bias, and zero to three has a very high risk of bias. In addition, the Cochrane method for assessing bias in non-randomized studied was used, which assessed for selective reporting, incomplete outcome data, adequate control of confounders, blinding, appropriate comparability of cohorts, appropriate sample size, and appropriate selection. A formal assessment of heterogeneity was not conducted as there was an insufficient number of high-quality studies with data suitable for a pooled quantitative analysis.

### Outcomes

The main outcomes of interest were the quality of the studies, the methods of weight estimation studied, and the accuracy of weight estimation by the studied methods (ideally percentage of estimates within 10% and 20% of actual measured weight [P10 and P20]). In addition, recurring themes arising in the included studies were identified and analyzed.

## Results

### Study Characteristics

A total of 25 studies which met the inclusion criteria addressing the subject of weight estimation in the prehospital setting were included (Table 1).^8–31^

**Table 1.** Studies Included in the Systematic Review^8–31^

Study	Method(s)	Study Design	Environment and Participants	N	Age Range	Findings/Limitations	Strength of Evidence (NOS)
Dieckman 1994 (USA)	Parent or Paramedic Estimates	Retrospective analysis of diazepam administration in status epilepticus	Prehospital Paramedics	36	Children <18 years	“In this study, 12 children, or 40% of all treated patients with status epilepticus, had a dose miscalculation. Based on hospital weights of the patients with status epilepticus, seven received less drug than the protocol minimum and five received more than the protocol maximum. A length-based method for drug dose calculations would be more accurate in the prehospital setting and would eliminate common paramedic errors in weight estimation.” The authors did not examine the relationship between weight estimation errors and treatment failure or adverse effects of medications. Many of the children were older than 12, the effective limit for the Broselow tape.	**
Martin 1994 (USA)	Paramedic Estimates	Retrospective record review	Multicenter Prehospital Paramedics	133	Adults	Paramedics were able to estimate weight within 10% in 74% and within 20% in 93% of cases, but methods used were unknown. “Current EMS training usually does not specifically address techniques for the assessment of patient weights.” Although an error of this magnitude could pose an increased risk of toxicity with pharmacologic agents commonly used in management of OHCA, this study suggests that scales or devices designed to assess patient weights are not needed on EMS vehicles as “these errors occur infrequently.” Only patients with ROSC were included. Large estimation errors and the resulting dose errors may have been more prevalent in non-ROSC patients.	*
Vilke 2001 (USA)	Paramedic Estimates, Broselow Tape	Prospective observational study – basic simulation scenario	Simulation Study Paramedics	20 medics estimated weight of four children and then used Broselow tape for one child (4.5kg)	4 children (4.5kg, 9.5kg, 10.5kg, and 17.3kg)	All methods were reported to be accurate with a mean error of 15%, but the Broselow tape was found to be the best. However, more than 10% of estimates had >50% error. “Use of the Broselow tape has helped to improve the accuracy of estimating the child’s weight, but not all prehospital programs are consistently using it.” Only part of the study focused on weight estimation, and there was no confidence interval or variance reported. Very wide margins of acceptable accuracy were used; limited evaluation of Broselow tape, drug doses calculated from tape in only one scenario.	****
Anglemyer 2004 (USA)	Paramedic Estimates	Prospective observational study	ED Paramedics	144 estimates by unknown number of paramedics	Adults >17 years	Weights estimated by multiple staff members in the ED, including a paramedic. Paramedics achieved a P5 of 21.5% and P20 of 82.6% - worse than doctors, nurses, and patients. “Our study showed that staff estimation of a patient’s weight is often inaccurate.”	****
Hall 2004 (USA)	Paramedic Estimates	Prospective observational study	ED Paramedics	Four paramedics performed 62 estimations	Adults >18 years	Weights estimated by multiple staff members in the ED, including a paramedic. “Patients were almost nine-times more likely to accurately estimate their own weight than providers were able to estimate the patients’ weight, OR 8.8 (5.1, 15.4)” Paramedics were significantly less accurate than doctors and nurses - accuracy within 5kg 19.4% vs 28.1%.	******
Kaji 2006 (USA)	Broselow Tape	Before and after study	Prehospital Paramedics	104 children before 41 children after	Children ≤12	Before: “Only 29 of 104 subjects in the 1994 to 1997 cohort received the correct dose, whereas 46 of 104 subjects received a first dose within 20% of the correct dose.” After: “Twenty-one of 37 subjects received the correct dose, whereas 24 of 37 subjects received a dose within 20%.”	**
Williams 2010 (Australia)	Paramedic Estimates	Prospective observational study	Simulation study - visual estimates from images Paramedic Students	234 paramedic students estimated weight of seven simulated patients	2 children aged 4 years and 6 years 5 adults	Students were found to not be able to accurately estimate weights using the visual estimation method. Estimates were less accurate in children than adults. P10 39.5% P20 73.2%	**
Heyming 2012 (USA)	Broselow Tape	Prospective cohort study	Prehospital Paramedics	466	Children <145cm in length	Medics were able to accurately use Broselow tape to determine weight, although under-estimated in patients over 30kg. Study did not control for additional factors. Limited data analysis and presentation. Some very large errors in weight estimation. Results mostly relied on correlation analysis, which can be misleading. Conclusions not entirely supported by the data. Notable differences between ED Broselow and paramedic Broselow results.	***
Hoyle 2012 (USA)	Broselow Tape	Retrospective record review	Prehospital Paramedics	230	Children ≤11 years	“We studied errors in administering six EMS medications commonly given to children: albuterol, atropine, dextrose, diphenhydramine, epinephrine, and naloxone. Medication dosing errors occurred in 125 of the 360 drug administrations (34.7%; 95% CI: 30.0, 39.8).” “Medications delivered in the prehospital care of children were frequently administered outside of the proper dose range when compared with patient weights recorded in the prehospital medical record.” Obese and underweight children were excluded, older children excluded. Broselow Tape weight was used as the standard, rather than measured weight.	***
Lammers 2012 (USA)	Broselow Tape	Prospective observational study	Simulation Study Paramedics	45 simulation sessions with single scenario	6-month-old infant (manikin)	Only 80% of crews were able to correctly use the tape, but there was a 60% error rate in drug dosing, partly due to inaccurate weight estimation (errors >10%). In addition, 20% of crews used inappropriate methods of weight estimation (all were inaccurate). Simulations only included weight estimation as a portion of the study – errors were identified but not closely examined.	**
Lim 2013 (USA)	Paramedic Estimates	Retrospective record review	Prehospital Paramedics	199	Children <18 years	EMS providers were found to accurately estimate weight but no information on the methods used to estimate was found (eg, whether parents were asked or whether it was an estimate from the paramedic). P20 82.4% led to dosing errors in 1/3 of cases. Large number of patients had no weight estimations and were excluded. Reference weight was not always measured at the time of admission.	**
Lammers 2014 (USA)	Broselow Tape	Mixed Method Study	Simulation Study Paramedics	142	5-year-old child (manikin)	A total of 35 of 37 groups that used the Broselow tape obtained an accurate weight, but there was still a 61% error rate in calculation for epinephrine dosage. The authors did not state how an accurate weight estimation was defined. Weight estimation not evaluated, other than to note that some providers did not use the Broselow tape and 2/37 used it incorrectly.	**
Campagne 2015 (USA)	Standard Methods, Broselow Tape	Two treatment crossover trial	Simulation Study Paramedics	20 paramedics	6-month-old infant (manikin) 1-year-old toddler (manikin)	The Broselow tape was found to be more accurate for drug doses than standard methods of estimating weight and drug doses. Small study. Weight estimation accuracy not directly evaluated.	****
Chassee 2016 (USA)	Caregiver Estimates via Emergency Medical Dispatch, Paramedic Estimates	Prospective case series	Prehospital 911 Call Operator	197 patients	Children ≤12 years	Dispatchers were able to accurately obtain weight estimates in children under two years, but accuracy fell off sharply as children got older. P20 of 82.2% under 2 years, 68.2% over 8 years. EMD P20 accuracy 83.8% in children <3 years; paramedics 81.8% EMD P20 accuracy 78.8% in children 3 to 7 years; paramedics 84.6% EMD P20 accuracy 68.2% in children ≥8 years; paramedics 75.0% Method of weight estimation: estimate of weight by paramedics 27.2%, family estimate 60.0%, Broselow tape 2.0% Only assessed patients under 12, and was only accurate if parental guess was accurate, many exclusions, not known how many parents not able to provide estimate.	*****
Rappaport 2016 (USA)	Broselow Tape, Handtevy Tape	Prospective Randomized Trial	Simulation Study Paramedics	80 paramedics 320 simulations	1-year old child (manikin) 5-year-old child (manikin)	Handtevy tape was found to be more accurate when giving dextrose, but both were the same for epinephrine. Incorrect use of tape 16.3% for Broselow and 8.7% for Handtevy. Simulation setting and weight estimation not specifically evaluated.	*****
Shah 2016 (USA)	Not Specified	Retrospective record review	Prehospital Paramedics	250 patients	Children 0 to 18 years	Analysis of management of actively seizing pediatric patients. Weight documented in only 36% of patients. No appropriate comparison between weights from EMS and from ED (correlation only). Dosing errors >20% in administration of midazolam in 42% of cases and errors in 49% of cases. Weight estimation method not reported - either parental estimate or paramedic estimate.	**
Hollis 2017 (Australia)	Not Specified	Retrospective record review	Prehospital Paramedics	153	All	Prehospital administration of ketamine: only 63% of patients had weight recorded. Weight estimation practices were a problem in this study. Need a better way of estimating weight (especially adults). No objective assessment of weight estimation accuracy	*
Wells 2017 (South Africa)	PAWPER XL tape	Prospective cohort study	Simulation Study - Visual Estimates from Images Paramedics and Paramedic Students	32 paramedics 960 estimations	Children 0 to 18 years	Study evaluated different methods of assessing body habitus as part of the PAWPER XL tape methodology. Paramedics assessed habitus as well as doctors, and better than nurses. Their weight estimations with the PAWPER method were accurate, with P10 71.7%, and P20 96.1%. Real children were not used, only photographs, performed in controlled setting rather than in a clinical setting.	********
Kaufman 2018 (Germany)	PaedER	Before and after	Prehospital Paramedics	59 before 91 after	Children 0 to 18 years	Dramatic reduction in errors after introduction of system. Not clear whether from improved weight estimation or from dosing information on the tape. Poor recording of weight in before group (0.5%). No specific reporting of weight estimation accuracy.	*
Wells 2018 (South Africa)	PAWPER XL Tape, Broselow Tape, Mercy Method	Prospective cohort study	Simulation Study Paramedics	8 paramedics 235 estimations	8 simulated patients - children from age 0 to 18 years	PAWPER XL MAC was the most accurate (P10 73.0% P20 95.2%), followed by the Mercy method (P10 57.3% P20 85.8%), and the Broselow tape (P10 47.7% P20 65.6%). Paramedics no different to other HCPs in accuracy using these methods. Limitations – simulation study, but with real child models.	********
Boehringer 2020 (USA)	Not Mentioned	Retrospective record review	Prehospital HEMS Crew	502	All	As GCS score decreased, the accuracy of weight estimations also decreased. Very poor methodology and data reporting. No description of outcomes. No data on accuracy. No description of reference method. The methods used to calculate weight were not known, inappropriate exclusion criteria introduced significant bias.	*
Hoyle 2021 (USA)	Parental Estimates, Age Formulas, Broselow Tape	Simulation study	Simulation Study Paramedics	Four different scenarios, 142 completed by 36 crews	1-month old (manikin) 6-month-old (manikin) 18-month-old (manikin) 5-year-old (manikin)	“[S]tudy of simulated pediatric EMS encounters, drug-dosing errors attributable to weight estimation were most frequent and demonstrated the greatest magnitude of error with use of patient age, were less frequent with BLT use, and least frequent with asking the parent for the patient’s weight.” Crews used own drug bags, choice of weight estimation and equipment. Many asked for parental weight estimate (51/142), but then used another method for dose calculation. For dosing: 12.1% parental estimates, 63.1% Broselow, age formula 24.8%. Accuracy of dosing much lower with age-based estimates BUT no actual, realistic weight was used as a standard – manikins were used. Substantial number of errors with Broselow tape use. Weight estimation errors (defined as >20% error): 1 parental estimate (no pound to kg conversion), 9 with age formulas and 8 with Broselow tape. Weight estimation accuracy not truly evaluated.	*
Kaufman 2021 (Germany)	PaedER	Before and after	Prehospital Paramedics	59 before 443 after	Children 0 to 18 years	Documentation of patient’s weight increased from 3.2% in 2007/2008 to 30.5% in 2018/2019. The overall rate of drug dosing errors decreased from 22.0% to 9.9%. No specific analysis of weight estimation.	***
Rappaport 2022 (USA)	Broselow Tape, Parental Estimates, Paramedic Estimates, Handtevy Tape	Before and after	Prehospital Paramedics	483 drug admin-istrations in 375 children	Children 0 to 13 years	Doses were correct with Handtevy system in 89.4% of cases, compared to 51.1% in baseline period. However, authors contended that Broselow LBT more closely approximates weight compared to Handtevy LBT when evaluated using national survey data. Therefore, improvement in dosing accuracy was probably not related to improved weight estimation accuracy. Weight estimation accuracy was not specifically studied.	
Ward 2022 (USA)	Not Specified	Retrospective record review	Prehospital Paramedics	3618	Children 0 to 14 years	Nearly 50% of weight-based medications given without a formal weight estimate. Error rate of 23% to 53% depending on age. Higher errors when pounds estimated. Used age-based formulas as a gold standard for comparison. Very poor methodology. No specific assessment of weight estimation accuracy.	****

Abbreviations: NOS, Newcastle Ottawa Scale (score in stars, from 0/worst to 10/best; P10, percentage of estimates within 10% of actual weight; P20, percentage of estimates within 20% of actual weight; GCS – Glasgow Coma Scale score; EMS, Emergency Medical Services; ED, emergency department; EMD, emergency medical dispatcher; HEMS, helicopter Emergency Medical Services; ROSC, return of spontaneous circulation; OHCA, out-of-hospital cardiac arrest; HCP, heath care provider.

Studies were conducted from 1994 through 2022. Most studies originated from the United States (19/25; 76.0%), with two studies (8.0%) originating from Australia, two (8.0%) from Germany, and two (8.0%) from South Africa. Twenty studies (80.0%) were pediatric weight estimation studies, three (12.0%) were adult weight estimation studies, and two studies (8.0%) included patients of all ages. There were 13/25 (52.0%) prospective studies, 8/25 (32.0%) retrospective record reviews, and 4/25 (16.0%) before-and-after studies. Of the 14 prospective studies, 9/14 (64.3%) were simulation studies. Five of the simulation studies used manikins, two used photographic images of children, and two used children as simulated patients. There were five clinical prospective studies (5/14; 35.7%), of which three were conducted in the prehospital environment, and two in the emergency department (ED; with paramedic estimators). The Broselow tape was evaluated in 12/25 studies (48.0%), paramedic estimates in 6/25 studies (24.0%), caregiver estimates in 4/25 studies (16.0%), other methods in 9/25 studies (36.0%), and unspecified methods in 5/25 studies (20.0%). Weight estimation accuracy was the primary objective of only 12/25 (48.0%) studies, while drug dosing accuracy was the primary objective in 13/25 (52.0%) studies.

### Quality of the Studies

On the Newcastle-Ottawa scale, 16/25 studies (64.0%) were at very high risk of bias, 6/25 studies (24.0%) were at moderate risk of bias, and 3/25 (12.0%) of studies were high-quality studies. The results of the Cochrane assessment of risk of bias are shown in Figure 2a and Figure 2b. The major risks of bias in the identified studies were incomplete reporting of outcome data, inadequate control of confounders, and weak study design or selection methodology.

**Figure 2a.**
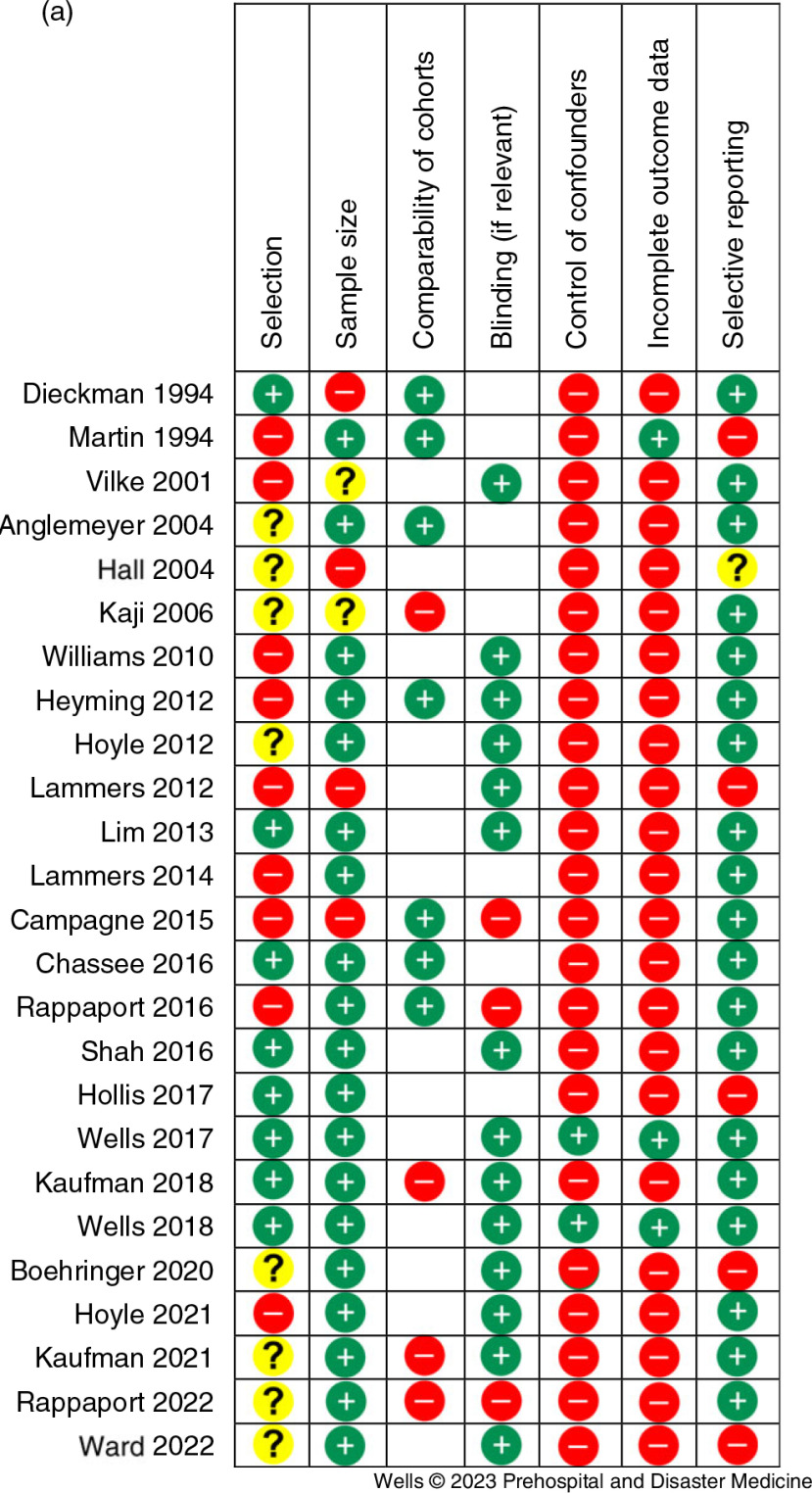
Quality Assessment for the Included Studies. Note: The Cochrane grading for each individual study is shown.

**Figure 2b.**
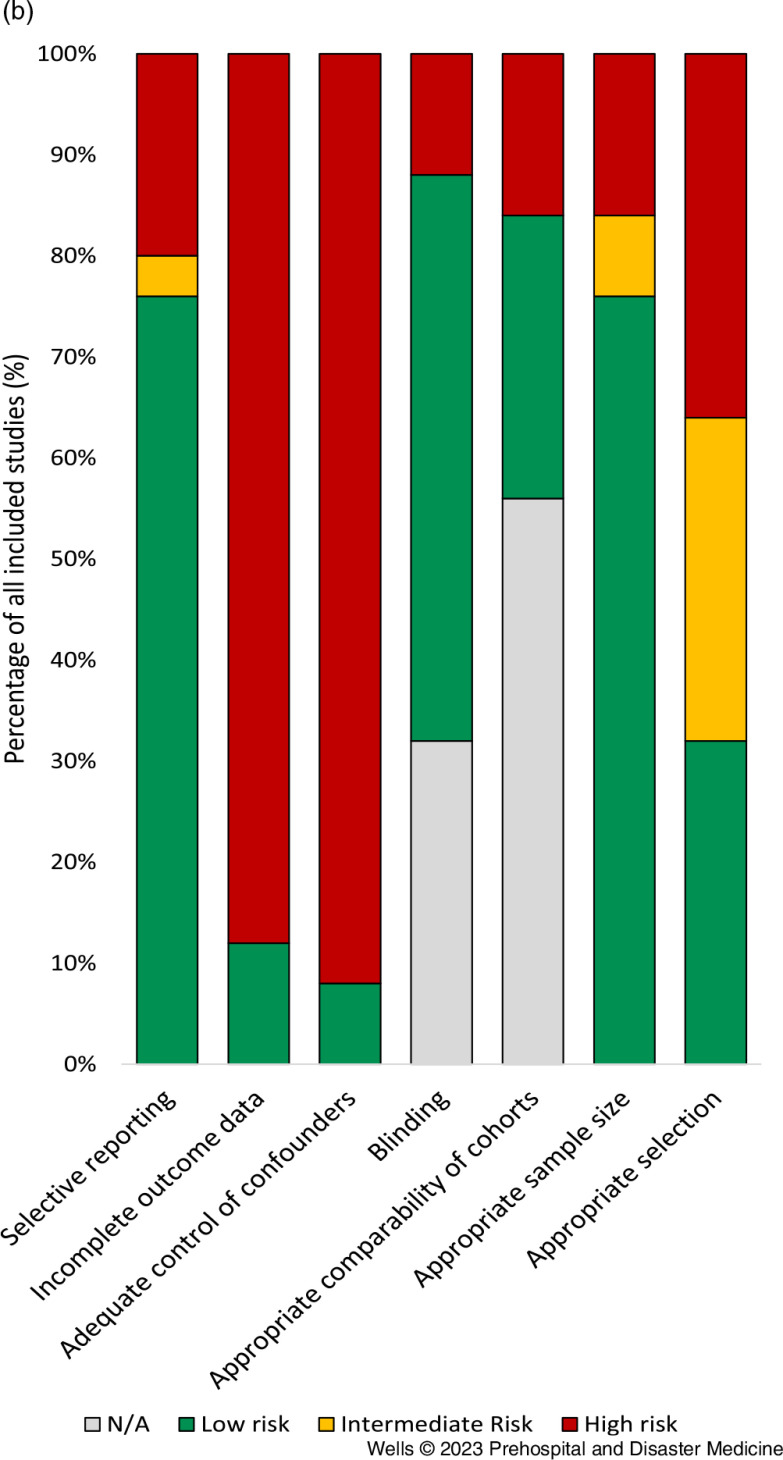
Risk of Bias Assessment for the Included Studies. Note: The cumulative scores for each category of risk are shown.

### Accuracy Outcomes

The data on the accuracy outcomes are shown in Table 2. Only nine (9/25; 36.0%) studies presented data that were helpful to evaluate the performance of the weight estimation systems: the Broselow tape (two studies), paramedic estimates (six studies), caregiver estimates (one study), unknown method of estimation (two studies), PAWPER XL tape (two studies), and the Mercy method (one study). The remainder of the studies (16/25; 64.0%) evaluated the combined effects of weight estimation and drug dose calculations on the final drug dose accuracy.

**Table 2.** Accuracy Data for Weight Estimation Systems

Method	Findings	Comments	Source
**Broselow Tape**	^c^P50 100%	Very wide acceptable dose range. Very large dose errors in 10% of patients, even with the use of the tape.	Vilke 2001
	^c,e^P20 57% (Dose Accuracy)	Improved from P20 44% (dose accuracy) in the before group. BT was used in approximately one-half of the before group.	Kaji 2006
	^c,e^No Interpretable Data	Only correlation analysis between prehospital and ED weights. Some large discrepancies between prehospital and ED BT weights.	Heyming 2012
	^c,e^P20 63.3% (Dose Accuracy)	This was probably primarily an inaccuracy of dose calculation rather than weight estimation, based on the reported methodology.	Hoyle 2012
	^d,e^P10 40% (Dose Accuracy)	BT often used incorrectly.	Lammers 2012
	^d,e^P10 95% (Dose Accuracy)	Still as much as 61% error rate in dose calculations.	Lammers 2014
	^d,e^P25 100% (Dose Accuracy)	No dosing errors with BT.	Campagne 2015
	^d,e^P20 36.2% (Dose Accuracy)	Tapes frequently used incorrectly.	Rappaport 2016
	^e^No Interpretable Data	Only correlation analysis between prehospital and ED weights.	Shah 2016
	^c^P10 47.7% ^c^P20 65.6%	Paramedics had the same accuracy as doctors.	Wells 2018
**Paramedic Estimates**	^a^P10 74.4%, ^a^P20 93.2%	Estimates in pounds – errors in conversion to kilograms not considered in this study.	Martin 1994
	^c^P50 82.5%	Very wide acceptable accuracy range.	Vilke 2001
	^a^P5 21.5%, ^a^P20 82.6%	Worse estimates in patients >90kg. Patient self-estimate errors were “rarely” >20%.	Anglemeyer 2004
	^a^Within 5kg 19.4%	Paramedics significantly less accurate than doctors and nurses. Patient self-estimates were much more accurate than paramedics’.	Hall 2004
	^b^P10 39.5% ^b^P20 73.2%	Weight estimates worse in children than in adults.	Williams 2010
	^d,e^P20 0%	No estimates were accurate.	Lammers 2012
	^c^P20 75.0 to 81.8%	Weight estimation slightly less accurate in older children (≥8 years).	Chassee 2016
**Caregiver Estimates**	^c^P20 68.2 to 83.8%	Weight accuracy substantially less accurate in older children (≥8 years).	Chassee 2016
**Paramedic/** **Parent/Self-Estimate**	^d,e^P20 87.3%	Obtaining an incorrect weight led to a drug dosing error in 18/142 (12.7%).	Hoyle 2021
	^c,e^Significant Dosing Errors in 40% of Children	Source of error may have been weight estimation or calculation errors.	Dieckman 1994
	^c^P20 82.4%	Dosing errors in 1/3 of patients with weight estimation errors.	Lim 2013
	^b,e^No Interpretable Data	Decreasing accuracy of estimates with decreasing GCS.	Boehringer 2020
**PAWPER XL tape**	^c^P10 71.7% ^c^P20 96.1%	Paramedics had the same accuracy as doctors, better than nurses.	Wells 2017
	^c^P10 73.0% ^c^P20 95.2%	Paramedics had the same accuracy as doctors.	Wells 2018
**Mercy method**	^c^P10 57.3% vP20 85.8%	Paramedics had the same accuracy as doctors.	Wells 2018

Notes: ^a^Studies in adults only; ^b^Studies in adults and children; ^c^Studies in children only; ^d^Studies in manikins; ^e^Studies in which the primary focus was on drug dosing accuracy, significant confounders for inferences about weight estimation accuracy.

Abbreviations: P10, percentage of estimates within 10% of actual weight; P20, percentage of estimates within 20% of actual weight; P50, percentage of estimates within 50% of actual weight; GCS, Glasgow Coma Scale score; BT, Broselow tape; ED, emergency department.

### Major Themes

The major themes identified in the included articles, related to paramedic weight estimation, are shown in Table 3.

**Table 3.** Major Themes Identified from the Included Studies

Major Theme		Reference
**1**	Correct drug dosing has an important impact on safe and effective drug treatment.	Dieckman 1994, Anglemeyer 2004, Hall 2004, Kaji 2006, Lim 2013, Lammers 2014, Campagne 2015, Wells 2017, Kaufman 2018, Wells 2018, Hoyle 2021, Kaufman 2021, Rappaport 2022, Ward 2022
**2**	Drug dose calculation errors and weight estimation errors are both important contributors to dosing errors.	Kaji 2006, Hoyle 2012, Lammers 2012, Lim 2013, Lammers 2014, Chassee 2016, Rappaport 2016, Shah 2016, Kaufman 2018, Wells 2018, Boehringer 2020, Hoyle 2021, Kaufman 2021, Rappaport 2022, Ward 2022
	Training is important to ensure accurate weight estimation.	Hoyle 2012, Lammers 2012, Lim 2013, Lammers 2014, Rappaport 2016, Shah 2016, Wells 2017, Wells 2018, Hoyle 2021, Kaufman 2021, Rappaport 2022, Ward 2022
**3**	Paramedic “guesstimates” of weight are unreliable.	Anglemeyer 2004, Hall 2004, Kaji 2006, Williams 2010, Lim 2013, Chassee 2016, Kaufman 2018, Wells 2018, Hoyle 2021
	Length-based methods of weight estimation could eliminate errors of paramedic estimation.	Dieckman 1994, Martin 1994, Vilke 2001, Kaji 2006, Williams 2010, Heyming 2012, Campagne 2015, Chassee 2016, Kaufman 2018, Hoyle 2021, Kaufman 2021
	Erroneous use of length-based tapes is not uncommon.	Heyming 2012, Lammers 2014, Rappaport 2016, Wells 2017, Kaufman 2018, Wells 2018, Hoyle 2021
**4**	An accurate weight estimation method for incapacitated adults is needed.	Anglemeyer 2004, Hall 2004, Hollis 2017, Boehringer 2020

## Discussion

Medication errors during prehospital emergency care of children are known to be the among the most frequent and most severe.^15,25^ This error is caused by two factors: errors in estimating the weight and errors during the drug dose calculation process. However, weight estimation is not always given sufficient importance.^32,33^ This is true not only in children, but in adults as well, when weight-based drugs are administered.^5,34^ However, there remains a critical gap in knowledge concerning which weight estimation systems are the most accurate and appropriate for use in the prehospital setting.^35^ As the safety and effectiveness of potentially life-saving drug therapy relies on accurate weight estimation, it is crucial to address this gap in understanding. The significance of this study lies in its contribution to addressing and highlighting this knowledge gap and providing valuable insights into improving patient safety and care in the prehospital environment.

### Quality of the Studies

The current study highlights a significant gap in high-quality studies in EMS weight estimation. Despite its importance, the number of papers addressing this topic is limited, with only 25 identified, of which merely nine contained relevant data. This underscores the urgent need for further research in this area to improve the accuracy and reliability of EMS weight estimation, and ultimately enhance patient outcomes.

The study designs, sampling, and selection were found to be suboptimal. Many of the simulation studies relied on weight estimations from manikins or images rather than actual patients. These studies should only be regarded as preliminary reports, and the findings should not be generalized to clinical environments. Several of the clinical studies used a before-and-after methodology. This format is typically regarded as a low-quality study design, and the findings should also be regarded as hypothesis-generating rather than substantive.^36^ The lack of prospective clinical studies was notable and is indicative of the poor quality of the available evidence. Compounding these issues were the small sample sizes, and a preponderance of convenience sampling, further limited the studies’ power to provide meaningful conclusions.

The failure to describe potential confounders was also a major weakness. Most studies did not evaluate weight estimation accuracy in subgroups of body mass index/BMI (and age-groups in children). These two factors are known to have a significant impact on the accuracy of weight estimation methods. The failure to include this information makes it impossible to compare outcomes in different studies.

Some studies failed to specify the weight estimation methods used in the study. This dramatically limited the usefulness of the information obtained. There is limited value in studying weight estimation accuracy without this information.

Taken together, these findings highlight the need for more robust study designs, larger sample sizes, and the evaluation of relevant confounding factors in future investigations of weight estimation accuracy. Additionally, the standardization of weight estimation methods and data reporting across studies would greatly facilitate the comparison of results and the identification of best practices.

### Accuracy Data

Studies from the Emergency Medicine literature have suggested that an acceptable standard for a weight estimation system is to achieve 70% of estimates within 10% of actual weight (P10 ≤ 70%) and 95% of estimates within 20% of actual weight (P20 ≤ 95%).^4,37^ This is a standard which is generally achieved by the newer length- and habitus-based weight estimation systems (such as the PAWPER XL tape and the Mercy method), and which is seldom reached by other methods, including the Broselow tape.^2,4^ In this review, only the PAWPER XL tape used by paramedics achieved this benchmark in two studies, and paramedic estimates of adults’ weights came close in a single study.^4,9,26^ None of these were clinical, prehospital studies, however.

The Broselow tape was found to out-perform paramedic estimates by Vilke, et al.^10^ Unfortunately, the acceptable error range used in this study was very large (±50%), which makes it difficult to compare these results with those from other studies. The only other useful study on the Broselow tape showed a poor accuracy in children and adolescent simulated patients.^26^ From the studies that evaluated drug dosing accuracy with the Broselow tape, it is possible to infer that weight estimation was more accurate than paramedic “guesstimates” and age-based formulas, but there was no direct evidence presented in any study to support this. It is worth noting that the accuracy, and acceptability, of the Broselow tape has been questioned in the Emergency Medicine literature because of its inaccuracy in underweight and obese children.^2,4,38^

The accuracy of visual estimates of weight by paramedics was poor, with the exception of a single study by Martin, et al.^9^ It has been well-established in other research that visual estimates of weight are unreliable, and frequently very inaccurate. There is reasonable consensus that this method of weight estimation should not be used.^2^

Interestingly, the single study evaluating caregiver estimates of weight showed that these estimates were substantially less accurate than reported in previous studies in the Emergency Medicine and pediatrics literature.^2,4,20^ This was a pragmatic, real-world study in which 9-1-1 dispatchers obtained weight estimates from family members of sick children. This study is a useful warning that real-world scenarios may be very different to the typical settings used for weight estimation studies, and with substantially different results. This finding is significant, and important, because parental estimates of weight are considered to be the gold standard for weight estimates in children, but there have been few real-life studies to confirm this.

The only information on other methods of weight estimation showed that the PAWPER XL tape performed very well and the Mercy method moderately in studies by Wells, et al.^24,26^ Age-based formulas performed poorly and were only reported in a single study by Hoyle, et al.^28^

In this review, when compared against doctors and nurses, there was no convincing evidence that paramedics were better or worse at estimating weight using the Broselow tape, the PAWPER XL tape, or the Mercy method.^4,26^ However, one study did find that paramedics were significantly less accurate than doctors and nurses at visual estimation of weight in adult patients.^12^ This evidence from a single study is weak, however. Since visual estimation of weight is generally condemned as a poor method of weight estimation, the relevance is limited in any event.^39,40^

Many articles not included in this review have suggested that a particular weight estimation system would be suitable for use during prehospital care, without actually studying it in this environment (eg, Lubitz, et al and Park, et al).^41,42^ It is not clear whether generalizing weight estimation performance data from a different environment, such as the ED, to the prehospital environment would be valid. In the Emergency Medicine and pediatric literature, the most accurate weight estimation methods in children are currently the PAWPER XL tape and the Mercy method.^2,4^ While it is possible that these methods could be accurate in the prehospital environment, their usability (with a comprehensive drug dosing guide) during the circumstances of prehospital emergency care would need to be tested and confirmed.^43^

Many of the included studies focused on the accuracy of drug dosing as the key outcome, but generally ignored the impact of inaccurate weight estimation on drug dose accuracy. Both weight estimation and the drug dose calculation and administration process need to be accurate and easy-to-use to ensure accurate drug delivery.^33,34^ This concept needs to be included in future research.

### Themes

Four major themes were identified from the included studies. Firstly, the importance of accurate weight-based drug dosing was recognized as crucial for safe and effective emergency drug therapy in the EMS environment. This highlights the critical need for further research in this field. This has already been identified by patient safety organizations as a vital aspect of ED care, and applies equally to the prehospital environment.^44,45^ Secondly, both weight estimation and drug dose calculation were identified as key contributors to drug dosing errors, underscoring the critical importance of training to ensure these processes are conducted accurately and efficiently. This emphasizes the need for comprehensive guidance and on-going training for EMS personnel, using accurate weight estimation methods and guides to drug dose calculation. Thirdly, paramedic “guesstimates” of weight were deemed unreliable, with length-based tapes being a preferable alternative. However, these length-based tapes are not without their limitations, including incorrect usage and inaccuracies in underweight and obese children, as well as in older children. More accurate weight estimation methods should be considered. Lastly, weight estimation in adults has not been adequately studied, and there are currently no reliable methods for estimating weight in incapacitated adults in the EMS setting. This highlights a critical gap in the literature and the need for future research to develop accurate and reliable weight estimation methods for this population. Taken together, these findings emphasize the importance of accurate weight estimation and drug dose calculation in EMS, and the need for on-going research and training to improve patient outcomes and ensure optimal care in emergency situations.

## Limitations

There were some important limitations in this study. Most importantly, there were limited data to draw firm conclusions about prehospital weight estimation because of the limited number of studies and the poor quality of many of the included research articles. Several studies were found in abstract form only and could not be included in this review, indicating a potential publication bias in this area of study.

## Conclusion

Not enough is known about the practice of weight estimation in the EMS setting or by paramedics. That is the most important finding of this study. It was found that there was very limited published information available, and the quality of existing studies was suboptimal. Therefore, no real conclusions can be drawn regarding actual weight estimation practices, what methods are currently used, nor the performance of weight estimation systems in the EMS environment. This is a significant threat to patient safety in the EMS environment, and there is a critical and urgent need for high-quality research. Wide recognition was found in the literature on the importance of weight estimation accuracy, drug dosing accuracy, and training in these processes for EMS personnel. Future research should focus on real-world, high-quality clinical research identifying which methods of weight estimation are most accurate and easy-to-use, and which drug dosing guides are most accurate and easy-to-use in adults and children. In addition, appropriate training methods and protocols need to be developed and studied to determine how best to ensure competency by users, as well as adherence to best evidence practices in this regard.

## Supplementary material

For supplementary material accompanying this paper visit https://doi.org/10.1017/S1049023X23006027. click here to view supplementary material
